# Development of a Mobile-First Registry to Recruit Healthy Volunteers and Members of Underrepresented Communities for Alzheimer’s Disease Prevention Studies

**DOI:** 10.14283/jpad.2023.86

**Published:** 2023-06-28

**Authors:** R. Aggarwal, E. Sidnam-Mauch, D. Neffa-Creech, A. Plant, E. Williams, E. Shami, U. Menon, S. George, Jessica Langbaum

**Affiliations:** 1 Provoc, Washington, District of Columbia, USA; 2 School of Computing, Clemson University, Clemson, SC USA; 3 Sentient Research, West Covina, CA USA; 4 Marketade, Bethesda, MD USA; 5 University of South Florida Health College of Nursing, Tampa, FL USA; 6 Department of Preventive and Social Medicine, Charles R. Drew University of Medicine and Science, Los Angeles, CA USA; 7 Department of Community Health Sciences, UCLA, Los Angeles, CA USA; 8 Banner Alzheimer’s Institute, 901 E. Willetta Street, Phoenix, AZ 85006 USA

**Keywords:** Alzheimer’s disease prevention, underrepresented communities, participant recruitment registry, user research

## Abstract

**Background:**

Web-based participant recruitment registries can be useful tools for accelerating enrollment into studies, but existing Alzheimer’s disease (AD)-focused recruitment registries have had limited success enrolling individuals from underrepresented racial and ethnic groups. Designing these registries to meet the needs of individuals from these communities, including designing mobile-first, may facilitate improvement in the enrollment of underrepresented groups.

**Objectives:**

Evaluate the usability of a prototype mobile-first participant recruitment registry for AD prevention studies; assess users’ perceptions of and willingness to sign up for the registry.

**Design and Setting:**

Quantitative usability testing and an online survey; online setting.

**Participants:**

We recruited 1,358 adults ages 45–75 who self-reported not having a diagnosis of mild cognitive impairment, AD, or other forms of dementia (Study 1: n=589, Study 2: n=769). Black/African American and Hispanic/Latino participants were specifically recruited, including those with lower health literacy.

**Methods and Measurements:**

Study 1 measures the prototype’s usability through observed task success rates, task completion times, and responses to the System Usability Scale. Study 2 uses an online survey to collect data on perceptions of and willingness to sign up for the mobile-first registry.

**Results:**

Study 1 findings show the prototype mobile-first recruitment registry website demonstrates high usability and is equally usable for Black / African American, Hispanic/Latino, and White user groups. Survey results from Study 2 indicate that users from underrepresented communities understand the registry’s purpose and content and express willingness to sign up for the registry on a mobile device.

**Conclusions:**

Designing mobile-first participant recruitment registries based on feedback from underrepresented communities may result in more sign-ups by individuals from minoritized communities.

## Introduction

**U**nless there is a medical breakthrough, the number of people living with Alzheimer’s disease (AD) in the United States is projected to nearly double by 2060 (1). Beginning in 2030, the Hispanic/Latino population in the US is projected to have the largest increase in the prevalence of AD and AD-related dementias (ADRD), followed closely by Black/African American (Black) adults (2). Even though Black and Hispanic/Latino adults are more likely than White non-Hispanic adults to have AD or cognitive impairment from ADRD (3–6), these groups are underrepresented in AD studies (7, 8), limiting the generalizability of findings and the ability to identify factors that may be pertinent to specific populations (9).

Web-based participant recruitment registries have the potential to reach large numbers of participants efficiently and have helped overcome some obstacles to enrollment (10, 11). However, most enrollees in AD-focused recruitment registry websites are White, non-Hispanic women (12–14). Additionally, existing AD-focused registry websites are not explicitly designed for mobile interfaces. Mobile websites require substantially different designs than desktop sites (e.g., less text, little to no hypertext, vertical navigation, etc.). As smartphone use among older adults continues to grow (15), it becomes increasingly important to create effective mobile-first registry websites. A mobile-first approach prioritizes designing a website that is easy to use on a mobile device versus designing a desktop website and simply making it accessible on mobile browsers. Mobile-first design focuses on communicating crucial information efficiently and making it simple for users to complete target interactions-like completing a sign-up form-on smaller, touch-based screens.

The lack of mobile-first registries may hinder participant recruitment for AD studies. It may also create an obstacle for recruiting individuals from underrepresented communities. Although disparities in smartphone ownership persist for particular groups (16), rates of smartphone ownership are now similar amongst Black, Hispanic/Latino, and White adults (17). Even so, Black and Hispanic/Latino individuals rely more heavily on smartphones when seeking health information (17). Existing recruitment registries struggle to correct misperceptions about the risk of developing AD and effectively communicate who qualifies for prevention studies (18). When recruiting historically underrepresented populations, it is important to recognize these groups face unique barriers, including low literacy and lack of access to health education (19), lower digital literacy (16), and an “understandable and long-standing” mistrust of the medical establishment due to untrustworthy research and researchers (20); these obstacles are in addition to numerous other barriers to recruitment and retention (21). To recruit underrepresented communities more effectively for research studies, it is imperative to develop mobile-first registries that successfully communicate key information and foster trust.

This study is the second component of a larger project which aims to identify barriers and facilitators for the recruitment of healthy adults from underrepresented communities (22), to identify best practices for the design of mobile recruitment registries, and to apply these practices to develop and evaluate a prototype registry. It is important to note the differences between mobile-responsive (i.e., smartphone and tablet compatible) and mobile-first designs. A mobile-responsive site is not necessarily a mobile-first site. While mobile-first websites are mobile-responsive, these sites always respond to changes in display sizes and are designed with the understanding that people interact with mobile devices differently than desktops. The prior study (22) evaluated mobile-responsive registries in general. The present study leverages those findings to develop a prototype mobilefirst AD-focused recruitment registry website to increase enrollment among healthy volunteers and members of underrepresented communities. This prototype was developed through iterative qualitative usability testing with target users (i.e., 45–75-year-olds, including adults who identify as Black and/or Hispanic/Latino, as well as those with lower health literacy). To examine how a mobile-first, user-informed registry performs and to understand how underrepresented communities perceive such a registry, we assess the registry through a quantitative usability test and an online survey.

## Methods

We conducted a quantitative usability test (Study 1) and an online survey (Study 2) from October 2020 through December 2020 to evaluate a user-informed prototype of a mobile-first recruitment registry. All procedures were approved by the Western Institutional Review Board.

### Prototype Development

**Figure 1:**
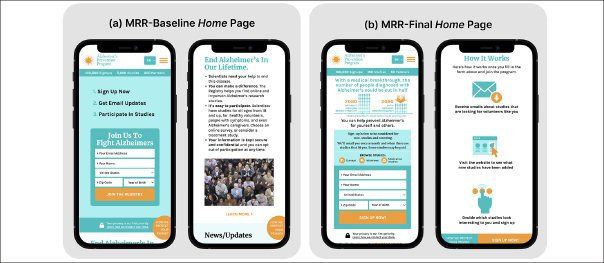
Screenshots of the home screens of (a) the Mobile-First Recruitment Registry (MRR) baseline prototype and (b) the final MRR prototype, developed through four rounds of qualitative usability testing with iterative prototyping

Prior to the studies, we developed a prototype registry, referred to as “Mobile-First Recruitment Registry (MRR)-Baseline”, applying design and messaging recommendations identified in prior research (18) (Figure 1a). We then conducted qualitative usability tests with iterative prototyping between rounds to develop the final prototype, referred to as “MRR-Final” (Figure 1b). We conducted four rounds of iterative prototype development and testing; in line with sample size recommendations for qualitative usability studies (23), we recruited six participants per round. In total, we enrolled 14 Black/African American and 10 Hispanic/Latino participants, a third of whom (n=8) reported lower health literacy according to the Single Item Literacy Screener (SILS) (24). All participants were healthy adults age 45 or older. Both males (n=11) and females (n=13) were enrolled.

Participants engaged in a 60-minute, semi-structured video interview where they interacted with the prototype and responded to questions about the site’s content and usability. Each testing round included Black and Hispanic/Latino participants, with at least one participant reporting lower health literacy. After each round of testing, two researchers (EW, ES) independently coded themes in the data. The findings were applied to iteratively improve the prototype’s design and messaging. By the fourth round of qualitative usability interviews, no usability issues were encountered, and all participants reported understanding the purpose of the registry and how to sign up. Figure 1 contrasts the homepages of the baseline and final MRR prototype.

### Study Procedures

To be included in the studies, participants needed to 1) be age 45 to 75, 2) self-report having access to the internet and a smartphone (and/or a tablet for Study 1); 3) self-report a race/ethnicity of Black and/or Hispanic/Latino (Study 1 also included White participants as a comparison group); and 4) self-report not having a diagnosis of cognitive impairment, such as mild cognitive impairment (MCI) or dementia due to AD. Participants were excluded if they could not speak, read, or respond to written survey items in English. Participants were compensated for completing study tasks.

Both studies recruited from online panels, which often include individuals who are more familiar with online surveys and technologies. Since we are creating a mobile-first registry, these online panels are appropriate for recruiting people who represent target users for the prototype. Stratified recruitment was used for both studies to ensure an approximately even distribution of participants across racial/ethnic groups. Study 2 recruited a subset of participants who also reported lower health literacy. Health literacy was measured as a binary variable (lower v. higher) based on responses to three items adapted from the validated four-item Brief Health Literacy Screening Tool (24). See (22) for more information.

#### Study 1: Quantitative Usability Assessment

We conducted a 2×3 between-subjects experiment to examine the impact of the prototype version (MRR-Baseline, MRR-Final) and race/ethnicity (Black/ African American, Hispanic/Latino, White) on reports of usability. Based on a priori power analysis in G*Power (25), our target sample was 600 respondents to be adequately powered (beta=.80, P=.05) to detect significant differences (odds ratio=1.72) between three groups. Participants were recruited through MTurk (26) and Prolific (27); users were recruited from within each race/ethnicity group across both platforms until a quota for each group was reached. Qualified participants were directed to an online testing platform (loop11.com), where they completed study tasks. Participants were shown either the MRR-Baseline or the MRR-Final and asked to complete the sign-up process using dummy information and respond to a usability questionnaire. Participants were also asked to explore the landing page for 30 seconds and rate the site’s trustworthiness using a single Likert item: “How trustworthy is this site?” (1=Very trustworthy, 5=Not at all trustworthy).

We assessed the dependent variable, usability, via three measures: task success rate (a binomial measure of whether participants were able to complete the registry sign-up process), task completion time (a ratio-level assessment of the time needed to complete the signup process, measured in seconds), and a 9-item System Usability Scale (SUS) (items 2 through 10 of the full SUS (28); the mean SUS score identified in the literature (29) is 69.69, SD=11.87).

SPSS version 27 was used for the analyses, with alpha set at ≤ 0.05. A two-way MANOVA was run with race/ ethnicity and prototype version as the independent variables and SUS score and sign-up duration as the dependent variables. The data were checked to ensure they met assumptions for MANOVA; since signup duration was non-normally distributed, a logtransformation was applied to the data to achieve normality. Multiple logistic regression was run to test differences across race/ethnicity and prototype variant on task success rate.

#### Study 2: Survey Assessment

**Figure 2:**
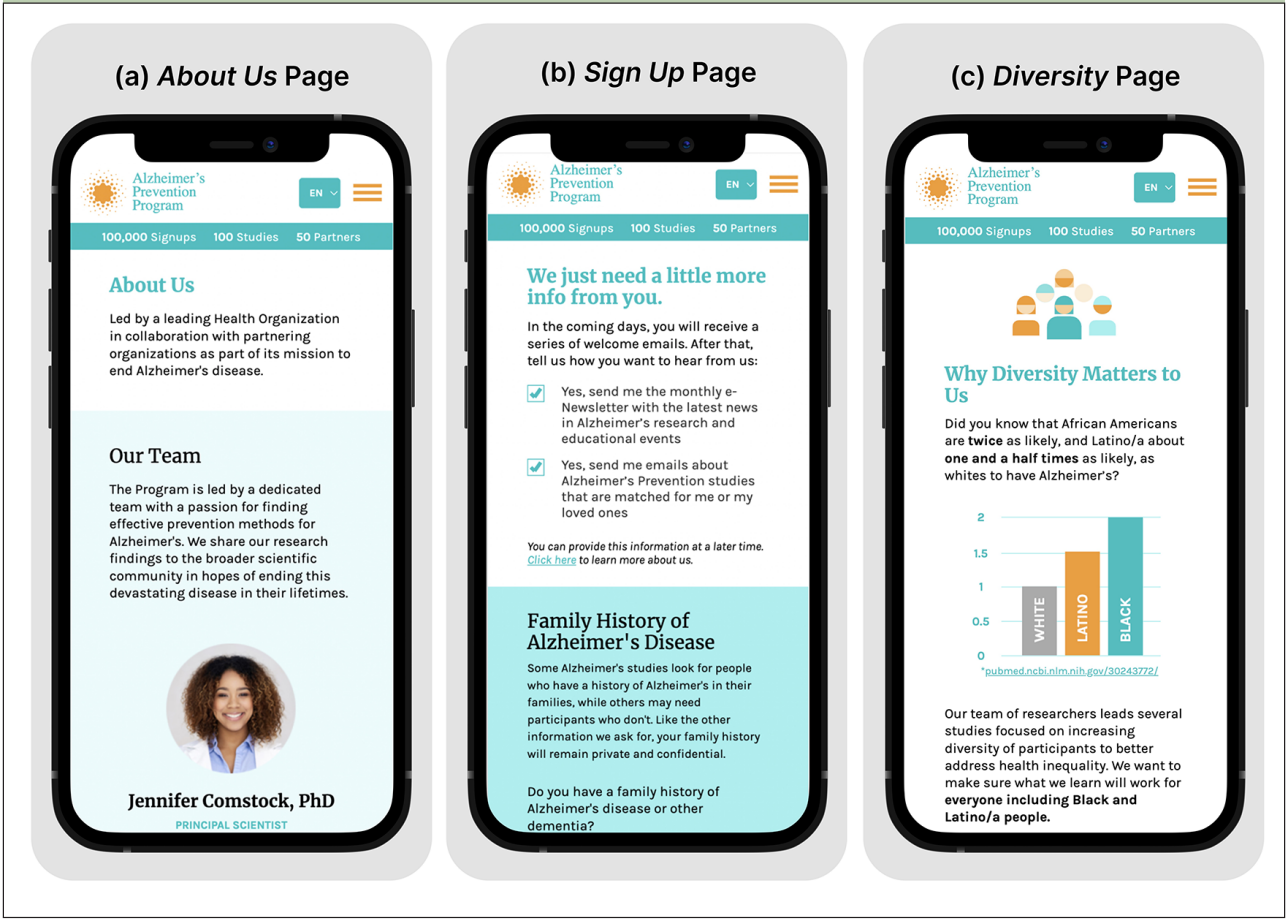
Screenshots of the top sections of the MRR-Final’s About Us, Sign Up, and Diversity pages. Participants could scroll down to see additional information on each page

We conducted an online survey to assess perceptions of the MRR-Final. Participants were recruited through a third-party panel service that maintains a database of 6 million individuals. Respondents from the Black/African American and Hispanic/Latino groups were randomly emailed a study invitation until a quota for each race/ ethnicity group was reached. Participants viewed the MRR-Final’s Home (Figure 1b), About Us (Figure 2a), Sign Up (Figure 2b), and Why Diversity Matters to Us (Figure 2c) pages and answered a series of questions about the understandability and relevance of the content and their likelihood of signing up for the mobile registry.

SPSS version 27 was used to analyze survey data. Responses were assessed with bivariate statistical analyses, namely, Chi-square tests and independent samples t-tests. Post hoc logistic regressions were run as well. For all analyses, we set significant p-values ≤ 0.05 using two-tailed analyses and checked that the data met statistical assumptions for each test.

## Results

### Participants

We recruited 1,358 healthy older adults across the two studies (Study 1: n=589, Study 2: n=769). Participant demographics for Study 1 and 2 are summarized in Table 1. Additionally, Table 2 provides a breakdown of the sample compositions from Study 1 and 2 by relevant demographics.

**Table 1:** Participant demographics for Studies 1 (Usability Experiment) and 2 (Online Survey)

**Variables**	**Study 1 (N=589)**	**Study 2 (N=769)**
	n (%)	n (%)
Race/Ethnicity		
Black/African American	197 (33.4)	399 (51.9)
Hispanic/Latino	195 (33.1)	370 (48.1)
White alone*	197 (33.4)	N/A
Age		
45–59	483 (82.0)	525 (68.3)
60–75	106 (18.0)	244 (31.7)
Lower Health Literacy	77 (13.1)	315 (41.0)
Sex (Female)	291 (49.4)	437 (56.8)

*“White alone” refers to participants who reported “White” as their race and did not report additional races/ethnicities. We excluded White individuals who also identified as Hispanic or biracial in order to compare the usability of the prototype for historically marginalized race/ethnicity groups (i.e., Black / African American and Hispanic/Latino).

**Table 2:** Breakdown of Study 1 and 2 samples by relevant demographics

**Breakdown of Study 1 (N=589) participant demographics by recruitment platform, race/ethnicity, and experimental condition**									
**Recruitment Platform**	**Race/Ethnicity**	**Assigned MRR Prototype**	**n**	**Gender**		**Literacy**		**Age**	
				**Men**	**Women**	**Higher**	**Lower**	**M**	**SD**
MTurk* n=414	Black/African American	Baseline	71	35	36	64	7	53.2	6.4
		Final	67	36	31	57	10	52.1	5.9
	Hispanic/Latino	Baseline	70	34	36	59	11	51.9	5.5
		Final	71	36	35	53	18	54.2	6.3
	White	Baseline	68	30	38	61	7	53.9	6.3
		Final	67	31	36	60	7	55.7	6.3
Prolific** n=171	Black/ African American	Baseline	27	13	14	25	2	53.0	7.0
		Final	32	14	18	29	3	54.2	7.5
	Hispanic/Latino	Baseline	30	16	14	27	3	50.9	5.0
		Final	24	12	12	20	4	54.8	8.6
	White	Baseline	30	20	10	27	3	51.3	6.5
		Final	32	21	11	30	2	51.8	5.6
**Breakdown of Study 2 (N=769) participant demographics by race/ethnicity**									
**Race/Ethnicity**	n	Gender***		**Literacy**		Age			
		**Male**	**Female**	**Higher**	**Lower**	**M**		**SD**	
Black/African American	399	172 (43.2%)	226 (56.8%)	234 (58.6%)	165 (41.4%)	56.2		7.01	
Hispanic/Latino	370	155 (42.3%)	211 (57.7%)	220 (59.5%)	150 (40.5%)	55.2		6.84	

* For MTurk, the following prescreeners were used for recruitment: Location (United States) and Age (“45 - 54” and “55 or older” categories were both selected). MTurk participants meeting the above criteria were sent to a screener questionnaire which collected information about their age, gender, and and race/ethnicity. For race/ethnicity, participants were asked «How would you best describe yourself?» and were provided 13 race/ethnicity options (single response); users met inclusion criteria if they selected “Black, African American”, “Hispanic, Latino, or Spanish origin”, or “White”. Users meeting the location, age, and race/ethnicity qualifications were randomly selected and sent a direct invitation to the study (via MTurk). **For Prolific, the following prescreeners were used for recruitment: Location (United States); Age (45 – 75); and Ethnicity (Black/African American, Latino/Hispanic, or White/Caucasian). To stratify the sample, a separate project was launched in Prolific for each race/ethnicity group, with the only difference in the project being the race/ethnicity selected in the inclusion criteria. ***5 participants identified as another gender, not included here

### Quantitative Usability Test Results (Study 1)

The mean SUS score for the MRR-Final was 84 (SD=16), which is 15 points higher than the reported average score for websites and technologies evaluated by the SUS (29). Most participants (92%) successfully completed the registry sign-up task (269 passed; 24 failed to visit the “Thank You” page that was displayed after the second page of the sign-up form was submitted). The average participant completed the MRR-Final registry sign-up in under two minutes (M=118s, SD=57s). The MRR-Final’s mean trustworthiness score was 2.01 (SD=.87), meaning the average participant rated it “trustworthy.”

There were no statistically significant differences in usability based on the prototype version or user group. The interaction effect between race/ethnicity and prototype version on the combined usability variables (SUS score and sign-up duration) was not significant, F(4, 1164)=0.142, P=.967, Wilks’ Λ=.999, partial η2=.000. Neither the main effect for the prototype version (F(2, 582)=0.568, P=.567, Wilks’ Λ=.998, partial η2=.002) nor the main effect for race/ethnicity (F(2, 582)=0.955, P=.431, Wilks’ Λ=.993, partial η2=.003) had a significant effect on the usability variables. A post hoc one-way MANOVA indicated that age (measured as a categorical variable; 45 – 59 and 60 – 75) did not have a significant effect on the combined dependent variables of Trustworthiness, Task Completion Time, and 9-Item SUS score F(3, 289)=0.423, P =.737, Wilks’ Λ=.996, partial η2=.004.

The logistic regression model was not statistically significant, χ2(4)=1.841, P=.765, indicating neither the prototype version nor participants’ race/ethnicity influenced task success rate. A post hoc logistic regression shows that age (continuous) did not impact task success rate either, exp(B)=1.053, P=0.89, 95% CI: .992, 1.117.

### Survey Results (Study 2)

After interacting with four of the MRR-Final’s mobile pages (see Figure 1b and Figure 2), nearly half (49.4%) of participants reported they would likely sign up for the prototype registry on their mobile devices. The other half was split between neutral (28.1%) and unlikely (22.5%). There were no differences in the likelihood of signing up for the MRR-Final based on race/ethnicity (X^2^(1)=.272, P=.602), health literacy level (X^2^(1)=.849, P=.357), or past participation in a health-related study (Yes v. No; X^2^(1)=1.06, P=.302). As shown in Table 3, more than half of the participants indicated they “agreed” or “strongly agreed” that the content on the MRR-Final pages would make them “more likely to sign up” for the registry website.

**Table 3:** Percentage who agree/strongly agree with statements about MRR-Final’s webpages, including results of any statistically significant differences between racial/ethnic/literacy groups

Statement*	**MMR-Final Screen**							
	**Home**		**About Us**		**Sign Up**		**Diversity**	
S1) The content on this screen is relevant to me	53.1%		58.5%		59.3%		61.3%	
S2) The content on this screen would make me more likely to sign up for the registry website	56.4%		58.1%		57.9%		58.9%	
S3) I understand all of the content on this screen	69.2%		73.2%		75.7%		75.4%	
**S3 t-test results†**	**Home**		**About Us**		**Sign Up**		**Diversity**	
t-tests results‡	t(585.75)=3.068 P=.002		t(600.97)=2.109 P=.035		t(594.04)=2.239 P=.026		t(592.73)=2.695 P=.007	
Cohen’s d	.233		.159		.170		.204	
Means and SD for higher (HHL)/ lower health literacy(LHL)	HHL n=454	LHL n=315	HHL n=453	LHL n=315	HHL n=454	LHL n=315	HHL n=453	LHL n=315
	3.97 (.84)	3.76 (1.03)	4.03 (.80)	3.89 (.95)	4.07 (.81)	3.92 (.98)	4.08 (.81)	3.90 (.97)

* Responses measured via a 5-point Likert scale (1=Strongly Disagree, 5=Strongly Agree); †t-tests were run to see if responses to statements differed by participant race/ ethnicity (Black / African American v. Hispanic/Latino) or health literacy (higher v. lower). The only significant differences (P<.05) found were in responses to the “I understand all the content…” item by health literacy; ‡The tests did not assume equal variances as the Levene’s Test was violated (P=.000) for each

Most participants (83.5%) indicated it was clear that the purpose of the registry is to have people sign up to receive information about current and future AD research study opportunities. A post hoc logistic regression using age (continuous) as the predictor of participant response (yes - purpose is clear v. no - not clear) indicates that age did not significantly impact whether participants thought the meaning of the site was clear, (exp(B)=.963; P=.101; 95%CI: .921, 1.007). In contrast, a post hoc Chi-square test (yes v. no; LHL v. HHL) shows that responses differed by health literacy category: χ2(1)=9.180, P=.002. Even so, agreement was high in both groups, with 88.5% of those with higher health literacy agreeing “yes” the site’s purpose is clear (7.5% were “unsure” and 4.0% indicated “no” it was not clear) and 79.0% of those with lower health literacy agreeing that the purpose is clear (12.1% “unsure”, 8.9% “no”).

More than half “agreed” or “strongly agreed” that they understood the MRR-Final’s content, that the content was relevant, and that the content would make them more likely to sign up (see Table 3). To explore whether these results differed by health literacy or racial/ethnic group, we conducted t-tests for all items in Table 3. The only statistically significant (P<.05) differences were that participants with higher health literacy were more likely than those with lower health literacy to understand the content of all pages (see Table 3); the effects ranged from small (Cohen’s d>.20) to negligible (Cohen’s d<.20). There were no significant differences between health literacy level and perceptions of the content’s relevance or perceptions of its likelihood to impact sign-up intentions (P>.05 for all tests). There were no significant differences (P>.05) between race/ethnicity and reactions to the MRR-Final content. Post hoc sensitivity tests show the t-tests were adequately powered (beta=.80, P<.05) to detect differences with small effects between the racial/ethnic and literacy groups (Cohen’s d =.202 and Cohen’s d =.206, respectively).

On the Diversity page, participants were shown images of diverse staff and were introduced to the statistic showing that AD affects Black/African American and Hispanic/Latino adults more than White adults (Figure 2c). A majority (63.3%) of respondents said they had not heard this statistic before; 10.1% were unsure whether they had heard it. Across all health literacy and racial/ ethnic groups, at least 85% of participants said it was “Somewhat important” or “Very important” to see diversity among program staff on the website.

## Discussion

Participant recruitment registry websites have the potential to efficiently connect volunteers with research studies. However, most AD-focused registry websites have not been successful enrolling people from traditionally underrepresented communities. In this study, we conducted a quantitative usability experiment and an online survey to assess the usability and perceptions of a mobile-first participant recruitment registry for AD-focused studies. We recruited healthy adults from traditionally underrepresented communities to assess the registry (i.e., Black/African American and Hispanic/Latino participants, including those with lower health literacy). Participants indicated the MRR-Final was easy to use (according to the SUS), and 92% successfully completed the sign-up process, which took just under two minutes.

Although digital divides in smartphone ownership have decreased (15, 17), minority racial/ethnic groups are more likely to use smartphones when seeking health information (17) while also facing barriers like lower digital literacy (16). Thus, it is meaningful that there were no significant differences in the prototype’s usability amongst the racial/ethnic groups, indicating the MRR-Final did not create usability barriers for Black and Hispanic/Latino participants compared to White participants. Furthermore, on average, participants rated the MRR-Final as trustworthy. There were no significant differences between the technical usability of the MRR-Baseline and the MRR-Final; this is unsurprising since the MRR-Baseline applied usability best practices.

Beyond technical usability, a participant recruitment registry must meet specific user needs to be successful. Prior focus group and survey research (18) found that a significant barrier to people joining a registry included skepticism about the medical field/industry and confusion about the purpose of research registries. Adding information to address these barriers may not impact the technical usability, but it may affect a person’s perception of the registry and the likelihood of joining. Thus, in Study 2, we assessed users’ perceptions of the MRR-Final and their self-reported likelihood of signing up for the registry. Over half of Black and Latino participants reported they understood the informational content on the MRR-Final and found it relevant. Most participants “agreed” or “strongly agreed” the content on each page would make them “more likely to sign up for the registry website”. These results were consistent across racial/ethnic and health literacy groups; the only significant difference identified was that higher health literacy participants were slightly more likely to report understanding the content.

There are some limitations to the present study. While most participants agreed or strongly agreed that MRR-Final’s informational content would make them “more likely” to sign up, in this developmental study, we did not test whether the MRR-Final increased signup numbers compared to existing AD-focused, desktop-based registries. It also remains unknown whether there would be differences in a registry member’s behavior (e.g., enrolling in studies) on the MRR-Final compared to registries that have not been designed as mobilefirst with input from target groups. While the mobilefirst prototype in this study showed high usability and received positive assessments, more research is needed to understand how a mobile-first approach and user-informed design strategies perform compared to a desktop-first design approach or registries designed without iterative prototyping based on user input. Future work should test the comparative effectiveness of these approaches, including testing whether the MRR-Final is more successful at facilitating registry and study sign-ups than existing registries.

Additionally, while the MMR-Final had high usability and task success rates, 24 of the 293 participants who interacted with the MRR-Final failed to click through to the “Thank you” page. Task success rate did not vary by race/ethnicity or age, indicating the sign-up process did not create barriers for relevant subgroups. It is possible participants skipped over the “Submit” button because they did not think it was necessary for the purposes of the survey, but future studies could do follow-up interviews with those who fail the sign-up task to identify how task success rate can be further increased. Similarly, 4.0% of participants with HHL and 12.1% with LHL indicated the purpose of the MRR-Final was not clear. A national, online survey of diverse older adults (29) found that general intention to join a research registry was low, and intention was higher when joining a registry was tied with performing a specific task. It may be that in the present study, the individuals who indicated the site’s purpose was not clear did so because they want to know the specific tasks required of them. Future work should examine how to further communicate the purpose of a registry, particularly to those with lower health literacy.

Another limitation of this work is that our results may not generalize to other underrepresented groups beyond English-speaking Black/African American and Hispanic/ Latino adults. Other registries, such as the CARE Registry (31), have been designed to meet the specific cultural and linguistic needs of their target audience. Additionally, this study focuses on developing a registry to help with recruitment of healthy participants for AD prevention studies, and thus we did not examine how people with memory and thinking problems or their caregivers would engage with a mobile-first registry. Future work could study our prototype or similar registries in other underrepresented groups, languages, and populations to further understand how to design mobile-first registries that are usable for all relevant groups.

## Conclusion

Currently, AD-focused participant recruitment registries struggle to recruit diverse populations. A mobile-first registry may help increase the enrollment of individuals from traditionally underrepresented communities. Through a quantitative usability experiment and an online survey, we obtained input from target users (i.e., healthy adults ages 45 to 75, particularly those who identify as Black or Latino) to evaluate a prototype mobile-first registry that was developed through iterative prototyping based on input from underrepresented racial/ethnic communities, including those with lower health literacy. The MRR-Final displayed high technical usability. Additionally, participants perceived the registry website as trustworthy. They indicated they understood the registry’s purpose and content, found it relevant, and would be likely to sign up. Thus, we recommend registry builders seek feedback from target populations throughout the development of a registry website to design for the needs of underrepresented communities.

The data from the MRR-Final suggests that mobilefirst registries may succeed at enrolling individuals from underrepresented communities. Our study is an important and practical step forward for increasing the diversity of enrollees in AD-focused participant recruitment registries by designing and assessing a mobile-first registry that considers feedback from underrepresented groups. While the MRR-Final prototype was created for the purposes of this study and does not currently exist as a live site, the design and assessment process used in this study and the sample content in the prototype screenshots may inform the development of participant recruitment registries for research on AD and other diseases. We recommend registry builders seek feedback from target populations throughout the development of a registry website to design for the needs of underrepresented communities. Our results suggest it may be possible to increase diversity in AD participant-focused registries if the needs of underrepresented groups are considered and integrated throughout the development of a mobile-first registry.

## Notes

*Ethical standards:* This study was approved by the Western Institutional Review Board and conducted in compliance with Good Clinical Practice guidelines, ethical regulations, and applicable local laws and regulatory requirements.
